# Improvement of swallowing‐related muscle mass assessed by ultrasonography in malnourished patient with Wallenberg syndrome: A case report

**DOI:** 10.1002/jgf2.428

**Published:** 2021-03-02

**Authors:** Hidetaka Wakabayashi, Masako Kishima, Masataka Itoda

**Affiliations:** ^1^ Department of Rehabilitation Medicine Tokyo Women's Medical University Hospital Tokyo Japan; ^2^ Department of Dentistry Wakakusa‐Tatsuma Rehabilitation Hospital Osaka Japan; ^3^ Department of Oral Rehabilitation Osaka Dental University Hospital Osaka Japan

**Keywords:** dysphagia, nutrition, rehabilitation, sarcopenia, stroke

## Abstract

We experienced a malnourished 62‐year‐old male patient with Wallenberg syndrome whose swallowing‐related muscle mass was shown to improve on ultrasound following aggressive nutritional therapy used to improve malnutrition. Dysphagia improved from no oral intake at admission to regular oral intake at discharge by aggressive rehabilitation nutrition. Rate increases in body weight, skeletal muscle index, and coronal cross‐section of geniohyoid muscle area assessed by ultrasound during the 131 days of hospitalization were 15%, 21%, and 33%, respectively. Aggressive nutritional therapy, aimed at improving malnutrition, can improve swallowing‐related muscle mass. Ultrasonography of the swallowing‐related muscles over time is useful in assessing dysphagia.

## Graphical abstract

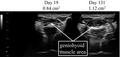

## 1 INTRODUCTION

In general and family medicine, there are many opportunities to examine patients with dysphagia, such as those who have suffered a stroke or aspiration pneumonia. For these patients, swallowing evaluation is important.^1^ The major causes of dysphagia are stroke, dementia, and sarcopenia. Ultrasonography is useful for assessing swallowing‐related muscles and swallowing function.^2^ However, there have been no reports on changes over time in swallowing‐related muscle mass using ultrasonography. We experienced a malnourished patient with Wallenberg syndrome (lateral medullary syndrome) whose swallowing‐related muscle mass was shown to improve on ultrasound following aggressive nutritional therapy used to improve malnutrition.

## 2 CASE

The patient was a 62‐year‐old man admitted to the hospital with dysphagia and ataxia. He was diagnosed with a dissecting aneurysm of the left vertebral artery using magnetic resonance imaging and angiography. Seven days later, he underwent trapping of the left vertebral artery and developed left Wallenberg syndrome. Twenty‐six days later, he was transferred to a convalescent rehabilitation hospital. The patient agreed to the publication of his case by providing informed consent.

## 3 ASSESSMENT AND TREATMENT

The patient's height and body weight were 175 cm and 54.1 kg, respectively, and his BMI was 17.7 kg/m^2^. Usual body weight for his height is 62 kg. He was diagnosed with malnutrition using the Global Leadership Initiative on Malnutrition (GLIM) criteria,^3^ which includes nonvolitional weight loss, low body mass index, reduced muscle mass, and reduced food intake. Total energy and protein intake at admission was 1,500 kcal given via nasogastric tube. The patient's skeletal muscle index (SMI) was 6.3 kg/m^2^, indicating low muscle mass, whereas hand grip strength was 29.6 kg, indicating normal muscle strength. He could not walk independently due to ataxia, not muscle weakness. Therefore, we diagnosed him as not having sarcopenia according to the Asian Working Group for Sarcopenia 2019 crieria.^4^

His dysphagia was severe; however, he showed a swallowing reflex once during a 30‐second repetitive saliva swallowing test. He scored a 1 (nothing by mouth) on the Functional Oral Intake Scale (FOIS).^5^ The cause of his dysphagia was considered to be bulbar palsy due to Wallenberg syndrome. He was not diagnosed with sarcopenic dysphagia because he did not have whole‐body sarcopenia.^6^ However, malnutrition might cause dysphagia, because he was diagnosed with malnutrition. Videofluoroscopy was performed on day 15. Dysfunction of hyoid bone excursion, laryngeal elevation and epiglottic inversion during swallowing were observed. He had marked pharyngeal residue in vallecula and insufficient opening of upper esophageal sphincter even after additional swallowing. He was diagnosed with bulbar palsy. The patient's motor and cognitive Functional Independence Measure (FIM) scores were 40 points and 35 points, respectively.

Physical therapy, occupational therapy, and speech therapy were performed 3 hours a day. The patient's total daily energy expenditure was 1950 kcal. This was based on a basal energy expenditure of 1300 kcal, calculated using the Harris‐Benedict equation, a gross activity factor of 1.5, and a gross stress factor of 1.0. His energy intake was planned to increase up to 2500 kcal with energy accumulation of 550 kcal to increase body weight by 2 kg/month, using aggressive nutritional therapy.^7^

On day 28, the patient started oral intake with soft food in combination with nasogastric tube feeding (FOIS 3; tube dependent with consistent oral intake of food or liquid). On day 45, he could eat 3 times a day and nasogastric tube feeding was stopped (FOIS 5; total oral diet with multiple consistencies, but requiring special preparation or compensations). His body weight was 56.0 kg, BMI was 18.3 kg/m^2^, and SMI was 6.9 kg/m^2^. On day 71, his FOIS was 7 (total oral diet with no restrictions). On day 107, his body weight was 59.3 kg, BMI was 19.4 kg/m^2^, and SMI was 7.2 kg/m^2^.

On day 131, the patient was discharged to home. His body weight was 62.5 kg, BMI was 20.4 kg/m^2^, and SMI was 7.6 kg/m^2^ (Table 1). He was no longer diagnosed with malnutrition, sarcopenia, and dysphagia. The patient's motor and cognitive FIM score were 91 points and 35 points, respectively, indicating independence in all activities of daily living.

**TABLE 1:** Timeline of the patient's clinical course

Day	1	19	37	51	72	92	107	121	131
Geniohyoid muscle area (cm^2^)		0.84	0.90	0.99	1.05	1.02	1.12	1.10	1.12
Body weight (kg)	54.1	53.7	54.6	56.0	57.1	57.9	59.3	61.0	62.5
Body mass index (kg/m^2^)	17.7	17.5	17.8	18.3	18.6	18.9	19.4	19.9	20.4
Skeletal muscle index (kg/m^2^)	6.3	6.7		6.9			7.2		7.6
Functional oral intake scale	1	1	3	5	7	7	7	7	7
Energy intake (kcal)	1500	2000	1867	2200	2200	2500	2500	2500	2500
Tongue pressure (kPa)	28.4	30.6				36.8			44.1
Handgrip strength (kg)	29.6	32.9				35.9			40.3

## 4 ULTRASONOGRAPHY OF A SWALLOWING‐RELATED MUSCLE

**FIGURE 1:**
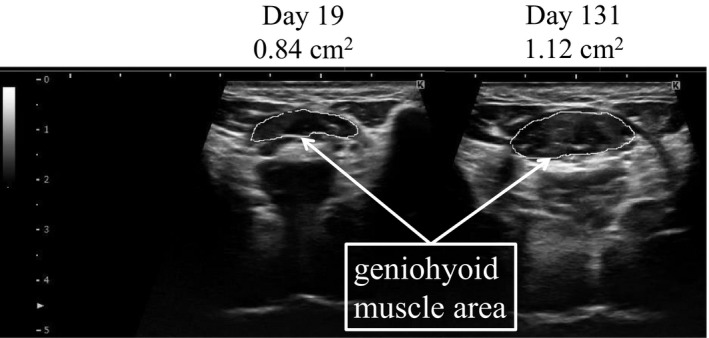
Coronal section of geniohyoid muscle area assessed by ultrasonography. Coronal section of geniohyoid muscle area increased by 33% from 0.84 cm^2^ at day 19 to 1.12 cm^2^ at day 131

A portable ultrasound (SONIMAGE MX1; Konica Minolta, Tokyo, Japan) with a linear and convex array transducer was used for the examination. The ultrasound examination was consistently performed by the same dentist (MK). The researcher placed the probe on the geniohyoid muscle, a swallowing‐related muscle located at 1/3 of the distance on a horizontal line between the parotid and the mandible^8^. A coronal cross‐section of geniohyoid muscle area (GMA) was assessed over time (Figure 1). GMA on day 19, 51, 103, and 127 was 0.84 cm^2^, 0.99 cm^2^, 1.12 cm^2^, and 1.12 cm^2^, respectively (Table 1). GMA increased in parallel with an increase in body weight and SMI. Rate increases in body weight, SMI, and GMA were 15%, 21%, and 33%, respectively. Echo intensity of geniohyoid muscle assessed by the ImageJ on day 19 and day 131 was 28.585 and 28.615, respectively.

## 5 DISCUSSION

This case study reveals that aggressive nutritional therapy, aimed at improving malnutrition, can improve not only body weight and appendicular muscle mass but also swallowing‐related muscle mass. Moreover, ultrasonography of swallowing‐related muscles over time is useful in assessing dysphagia.

Aggressive nutritional care management, aimed at improving malnutrition, can improve swallowing‐related muscle mass. Appendicular skeletal muscle mass and swallowing‐related muscles increased along with gains in body weight. This was due to rehabilitation and aggressive nutritional therapy that took into account energy accumulation as well as resistance training of the whole body and swallowing‐related muscles.^9^ Although the cause of the dysphagia in this case was stroke and not sarcopenia, the fact remains that lower muscle mass in swallowing‐related muscles has been associated with more severe dysphagia in acute stroke patients.^10^ We infer that the increase in geniohyoid muscle is mainly in muscle tissue, not fat tissue, because there was little difference in echo intensity between day 19 and day 131. Therefore, aggressive nutritional therapy is important for malnourished patients with dysphagia.

Ultrasonography of swallowing‐related muscles over time is useful in assessing dysphagia. Improvements in dysphagia may be due to an improvement in bulbar palsy, rather than an increase in swallowing‐related muscles mass. However, in cases of sarcopenic dysphagia, improvements can be due to increases in swallowing‐related muscle mass and strength. In clinical practice, deterioration of swallowing‐related muscle mass may be common in patients with aspiration pneumonia and sarcopenic dysphagia. In these cases, ultrasonography of swallowing‐related muscles over time may be used as a biomarker for dysphagia and sarcopenia.

There is a limitation to this case report. Time course and recovery of bulbar palsy by Wallenberg syndrome vary from patients. Dysphagia may be improved without aggressive nutritional therapy. However, swallowing‐related muscles mass does not increase considerably without aggressive nutritional therapy. Further studies are required to examine the effect of aggressive nutritional therapy on dysphagia in malnourished patients with Wallenberg syndrome.

In conclusion, aggressive nutritional therapy, aimed at improving malnutrition, can improve swallowing‐related muscle mass. Ultrasonography of the swallowing‐related muscles over time is useful in assessing dysphagia.

## CONFLICT OF INTEREST

The authors have stated explicitly that there are no conflicts of interest in connection with this article.
